# Engineering Granular Hydrogels without Interparticle Cross-Linking to Support Multicellular Organization

**DOI:** 10.1021/acsbiomaterials.4c01563

**Published:** 2024-11-25

**Authors:** Natasha L. Claxton, Melissa A. Luse, Brant E. Isakson, Christopher B. Highley

**Affiliations:** † Department of Biomedical Engineering, University of Virginia, Charlottesville, Virginia 22903, United States; ‡ Department of Molecular Physiology and Biophysics, University of Virginia School of Medicine, Charlottesville, Virginia 22903, United States; § Robert M. Berne Cardiovascular Research Center, University of Virginia School of Medicine, Charlottesville, Virginia 22903, United States; ∥ Department of Chemical Engineering, University of Virginia, Charlottesville, Virginia 22903, United States

**Keywords:** cellular connectivity, endothelial cells, fibroblasts, morphogenesis, granular hydrogels, microgels, permissiveness

## Abstract

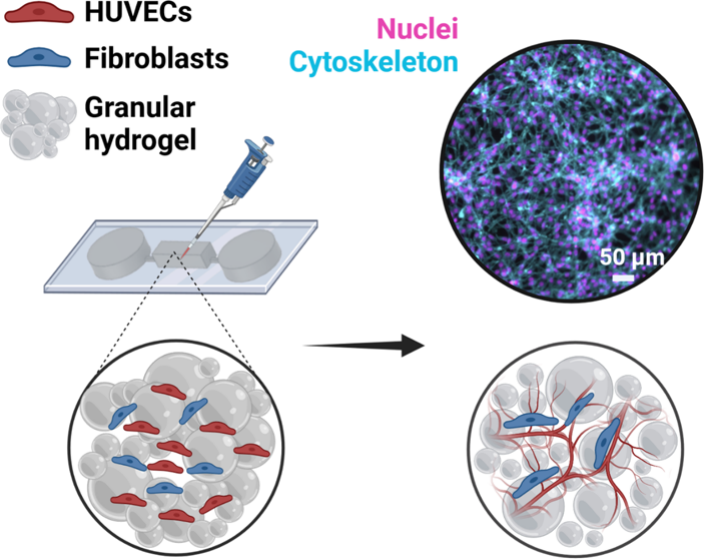

Advancing three-dimensional (3D) tissue constructs is central to creating *in vitro* models and engineered tissues that recapitulate biology. Materials that are permissive to cellular behaviors, including proliferation, morphogenesis of multicellular structures, and motility, will support the emergence of tissue structures. Granular hydrogels in which there is no interparticle cross-linking exhibit dynamic properties that may be permissive to such cellular behaviors. However, designing granular hydrogels that lack interparticle cross-linking but support cellular self-organization remains underexplored relative to granular systems stabilized by interparticle cross-linking. In this study, we developed a polyethylene glycol-based granular hydrogel system, with average particle diameters under 40 μm. This granular hydrogel exhibited bulk stress-relaxing behaviors and compatibility with custom microdevices to sustain cell cultures without degradation. The system was studied in conjunction with cocultures of endothelial cells and fibroblasts, known for their spontaneous network formation. Cross-linking, porosity, and cell-adhesive ligands (such as RGD) were manipulated to control system properties. Toward supporting cellular activity, increased porosity was found to enhance the formation of cellular networks, whereas RGD reduced network formation in the system studied. This research highlights the potential of un-cross-linked granular systems to support morphogenetic processes, like vasculogenesis and tissue maturation, offering insights into material design for 3D cell culture systems.

## 1 Introduction

Three-dimensional (3D) tissue constructs mimic complex aspects of tissue physiology and disease that cannot be modeled in two-dimensional (3D) environments.^1−3^ These constructs are used as *in vitro* models for providing further insight into research including engineering tissue replacements, drug discovery, and understanding physiology and disease.^1,4^ The development of tissue structures within 3D tissue constructs depends on cellular self-organization, which is influenced by material design. The control of biomaterial properties that influence these processes is critical for recapitulating the complexity of tissue physiology.^1,4^ The emergence of microvascular structures from cocultures of endothelial cells and pericytes,^5,6^ the differentiation of stem cells,^7^ and the development of tissue-like structures from organoids all depend on careful design of 3D materials.^8^ These processes often depend on designing environments that permit cell activity such as proliferation and movement, through either engineered degradation or permissive material properties including porosity and yielding to cell-applied stresses.^9−100^

Hydrogels that are permissive and capable of supporting the organization of 3D tissue structures include both natural and synthetic materials. Considering vascular morphogenesis as an example, where cells seeded in hydrogel develop into connected networks, robust 3D networks of endothelial cells form in fibrin^12^ hydrogels. Fibrin gels intrinsically support cell adhesion and remodeling and require no chemical modification to the polymeric backbone. Similarly, Matrigel is commonly used in assays for angiogenesis *in vitro.*(13,14) In moving toward advanced, synthetic hydrogel biomaterials used for tissue engineering^15,16^ that offer control over material properties, carefully designed chemical modifications that impart biochemical and biophysical functionalities and are used for cross-linking are advantageous. In these engineered materials, the hydrogel network structure can be designed to permit dynamic cellular activity within the hydrogel bulk. Approaches to engineering permissive synthetic systems include designing enzymatically degradable hydrogels,^17−19^ porous hydrogels, and hydrogels cross-linked with dynamic chemical bonds^6,21^ that all allow network rearrangement as cells proliferate and move in 3D.^22^

Particle-based (or granular) formulations of hydrogels^23,24^ can support 3D cellular activity without requiring degradable or dynamic chemistries in the hydrogel’s cross-linking or polymer backbone. A bulk granular hydrogel consists of hydrogel microparticles with inherent space between them ranging from a tightly packed granular structure to loosely packed.^15,16^ The porosity^25^ within a granular hydrogel^23,26−28^ provides potential for cellular movement and tissue morphogenesis. Granular hydrogel materials have drawn considerable attention in the field for tissue regeneration and 3D printing applications due to their size and inherent porosity for cellular infiltration.^21,24,29,30^ Seminal work with microporous annealed particle (MAP) hydrogels showed that their interparticle cross-linking, or annealing, supports tissue ingrowth and the formation of vascular networks from cells recruited from surrounding tissue when injected *in vivo.*(31) Work in the field has also shown that granular hydrogels that include cells, including vascular endothelial cells, between hydrogel particles^32^ foster an environment conducive to cell migration and proliferation^26^ in systems that include interparticle cross-linking.

Here, we examine the potential for granular, PEG-based hydrogels to support cell cultures in systems lacking interparticle cross-linking. Granular hydrogels without interparticle cross-linking,^23,33^ or annealing,^31^ possess inherent porosity and exhibit bulk yield stress behavior,^6,34^ both of which are beneficial. For example, when these materials are used for injection or in bioprinting,^24,31,35^ particle–particle interactions and jamming^28,36^ allow the granular hydrogel to maintain stability through interparticle cross-linking without being subjected to yield stress. In certain applications, this maintenance of yield stress behavior might be beneficial, for example, as a support bath for 3D printing. Granular hydrogels stabilized by jamming might also find uses in in vitro systems, where stress yielding behaviors like those in biological tissues^34,37^ might be desirable with respect to tissue growth and maturation,^38^ or with respect to facilitating dynamic cell behaviors.^23,36,39^ We therefore looked to understand whether these materials could support cellular expansion and the formation of cellular networks from cells in the absence of interparticle cross-linking. Because cocultures of cells used in vasculogenic^6,36,40^ systems are known to undergo dynamic organization to form networks in bulk hydrogels engineered to degrade,^41^ contain pores,^42^ or undergo stress relaxation,^6^ we used a coculture of endothelial cells and fibroblasts.

In this work, we investigated an endothelial cell and fibroblast coculture in 3D granular hydrogels formed from polyethylene glycol (PEG) microgels with intraparticle covalent cross-linking, but no interparticle cross-linking. PEG-based hydrogels are biocompatible, can be easily tuned to assess cellular behavior, and are used in many tissue engineering applications.^31^ We aimed to use a PEG particle population that approached cell scale (<50 μm average particle diameter) to facilitate a more homogeneous distribution of cells throughout the scaffold compared to larger particles. Both microfluidic^43−45^ and batch emulsification^46^ processes have been used to produce microparticle populations of this size, with polydispersity appearing in some high-throughput microfluidic systems^43,44^ and inherent to batch emulsification.^46^ Here, we expected an emulsion process to offer an accessible approach, scalable to milliliter-scale batches, for producing cell-scale PEG microgels and was scalable to milliliter-scale batches of particles that would retain desirable dynamic properties characteristic of well-defined colloidal-^47^ and granular-scale^48^ microgel populations. We established a culture device that could retain this material without degradation of particles from the granular system into the culture medium. Using these hydrogels, we then correlated the porosity and cell-adhesive ligands with the emergence of cellular networks within the material. We observed a rapid emergence of these networks, within hours, in un-cross-linked granular hydrogels. The approach may provide an alternative pathway for cellular networks and tissue structures and thus has broad potential application in tissue engineering and 3D cell culture systems.

## 2 Methods and Materials

### 2.1 Fabrication of PEG Hydrogel Microparticles (Microgels)

All reagents were purchased from Sigma-Aldrich unless indicated. PEG microgels were fabricated using a bulk emulsification method with a water-in-oil process. 6% (w/v) 4-arm PEG-acrylate (JenKem) and 4% (w/v) 4-arm PEG-thiol (JenKem) solutions were prepared in Dulbecco’s phosphate-buffered saline (DPBS). These solutions were mixed in a 1:1 ratio to give a final PEG concentration of 5% (w/v) with an excess of acrylates. For experiments that incorporated the arginine-glycine-aspartic acid (RGD) cell-adhesive ligand, 1 mg/mL thiolated RGD peptide (Arg-Gly-Asp) was included in the PEG hydrogel precursor solution to facilitate cell adhesion to the surface of the microgels. For fluorescence imaging of microgels, 1% (w/v) 2 MDa fluorescein-isothiocyanate-tagged dextran was included in the hydrogel precursor solution.

Light mineral oil including 1% (v/v) Span 80, and 0.5% (v/v) triethanolamine (TEOA) was prepared to form the continuous phase of the emulsion. The mineral oil solution was stirred at 2000 rpm using an overhead stirrer. The aqueous PEG solution was placed in a 1 mL syringe with a 30-gauge needle on a syringe pump. The aqueous phase was pumped at a rate of 40 μL/min directly into the stirred continuous phase to create a dispersion of aqueous droplets in the mineral oil. After the aqueous phase was fully dispensed into the oil, the water-in-oil suspension was allowed to stir for 1 h to allow the precursor solution to cross-link via Michael addition within the droplets, covalently incorporating thiolated RGD by the same mechanism when present.

Microgels were then recovered by transferring the suspension to a conical tube and centrifuging at 120 rcf for 5 min. Excess oil was removed, leaving behind a pellet of microgels. To wash the microgels, isopropanol was added to the conical tube, pipetted multiple times to mix, and then vortexed vigorously. The conical tube was then centrifuged again at 3200 rcf for 5 min to pellet the microgels, and the supernatant of isopropanol with residual oil was removed. The microgels were washed again with isopropyl alcohol and centrifuged two more times for a total of three isopropyl alcohol washes to ensure residual oil was fully removed. Finally, microgels were rehydrated with DPBS (1X) in the conical tube, vortexed, and centrifuged at 3200 rcf for 5 min. Excess DPBS was poured off, more DPBS was added in excess, and the mixture was centrifuged again. This process was repeated three times. After the last DPBS wash, fresh DPBS was added to the microgels at a 1:1 ratio with equal amounts of microgel volume to DPBS for storage in microcentrifuge tubes.

### 2.2 Formation of Granular Hydrogels

Before conducting cell experiments, stored microgels were centrifuged at 21,130 rcf to remove excess DPBS. Fresh endothelial cell medium (EGM-2, Lonza) was then added to the microgels at a 1:1 ratio, and microgels were allowed to equilibrate at least 24 h prior to experimentation. After equilibration, granular hydrogels were formed from the microgel suspension by centrifuging the microgels in microcentrifuge tubes at 21,130 rcf for 5 min. The endothelial cell medium supernatant was aspirated off the pellet to leave behind a fully jammed granular hydrogel. To create diluted granular hydrogels, endothelial cell medium was added at desired volumes to fully jam the granular hydrogels. The dilution ratios of granular hydrogel volume to cell medium volume used here, which changed the pore spacing between individual microgels, were 1:0 to 1:1 with 0.25 increments. Fully jammed granular hydrogels (1:0) were thus formed from microgels centrifuged at 21,130 rcf with no additional cell medium added. These particles were used for rheological experiments to assess granular hydrogel mechanical properties, in addition to microgel dilutions based on pore spaces used in cell culture.

When needed, granular hydrogels with interparticle cross-linking to limit the flow of microgels were formed by adding 66 mM photoinitiator, lithium phenyl-2,4,6-trimethylbenzoylphosphinate (LAP) to the granular hydrogels and photo-cross-linked at 10 mW/cm^2^ for 5 min to obtain a granular hydrogel in which particles were cross-linked to one another.

### 2.3 Granular Hydrogel Characterization

Granular hydrogel properties were characterized using rheological studies with a stress-controlled DHR-2 rheometer (TA Instruments), equipped with a 20 mm sandblasted parallel plate. Granular hydrogels were placed on the bottom plate of the rheometer. The top plate was rapidly brought down so that the gels formed a uniform disk between the rheometer plates. A solvent trap was used to prevent sample dehydration during the measurements. Mechanical tests, including stress relaxation and oscillatory tests, were performed once the storage modulus reached an equilibrium value. Stress relaxation tests were conducted between 4 and 20% strain. While the strain was held constant, stress was recorded over time. The stress relaxation time was normalized to the initial value. Oscillatory strain-dependent tests were conducted at a 0.1 to 1000% shear strain.

Porosity measurements were taken from confocal microscopy images (see below). Porosity was measured in the granular hydrogel formulations used in cell experiments. The test groups were the 1:0, 1:0.75, and 1:1 hydrogel:medium formulations, whose formation is described above. A high-molecular-weight (2 MDa) FITC-dextran was dissolved in the medium surrounding the microgels. Granular hydrogels were placed in a confocal dish and then imaged in digital z-stacks. Pore space was visualized and quantified through the fluorescence of FITC-dextran in the interstitial space surrounding the microgels in confocal slices. Images were processed in FIJI, thresholded, and pore space was analyzed.

### 2.4 Cell Culture

Human umbilical vein endothelial cells (HUVECs) were used for all experiments and cultured (passages 5–8) in an EGM-2 medium (Lonza). The culture medium was changed every 2 days. NIH 3T3 Fibroblasts were cultured (passages 10–13) in Dulbecco’s modified Eagle’s medium (DMEM/F-12) (Gibco) with 10% (v/v) fetal bovine serum and 1% (v/v) antibiotic/antimycotic solution. The medium was changed every 2 days. Cells were passaged when they reached 70–80% confluency.

For experiments using cocultures, HUVECs and fibroblasts were mixed at a ratio of 5:1 at concentrations of 5 million cells/mL and 1 million cells/mL, respectively, and combined with PEG microgels. Coculture experiments designed to observe the emergence of networks of cells were performed in EGM-2 medium supplemented with 100 ng/mL recombinant human angiopotetin-1 (Peprotech) and 50 ng/mL human VEGF-165A (ACROBiosystems). For transwell experiments, HUVECs and fibroblasts were mixed at a ratio of 2:1 due to the size of the transwell (6.5 mm) and stained after 4 days. Medium in the coculture environment was changed every 2 days. Cell morphology was observed after 24 h and 4 days. Images were taken at each time point.

### 2.5 Microdevice Design for Culturing Cells in Granular Hydrogels

In standard culture systems, granular hydrogels without interparticle cross-linking were observed to undergo surface loss during exposure to medium. A microdevice was designed to enable medium changes without dilution or loss of the granular systems studied. A polydimethylsiloxane (PDMS) device was used to contain the granular hydrogel during experiments with cells, and it was cast from a mold created using Autodesk Fusion360 and printed with a Formlabs Form2 3D printer. PMDS was prepared by mixing an elastomer base and curing agent at a 10:1 weight ratio. PDMS solution was placed under a vacuum to remove any air bubbles formed while mixing and was then poured into the mold. The mold was placed in a 37 °C incubator and left overnight for curing. Once cured, the PDMS device was removed from the mold and attached to microscope slides that had been plasma-treated. After being bonded to slides, devices were immersed in 70% ethanol for sterilization before use in cell studies.

### 2.6 Formation of Continuous PEG Hydrogels

Continuous PEG hydrogels were fabricated to match the granular formulation by combining equal volumes of 6% (w/v) 4-arm PEG-thiol and 4% (w/v) 4-arm PEG-acrylate solutions prepared in DPBS. We pipetted 500 μL of the PEG precursor solution into each well of a 24-well plate to cover the entire bottom surface and allowed it to sit at room temperature for a few hours until gelation. We seeded cells on top of the PEG hydrogels either with or without RGD, which was added as described in formulating microgels. Gels seeded with cells were centrifuged at 200 rcf for uniform cell distribution, and an endothelial culture medium was added to each well.

### 2.7 Formation of Cell-Containing Fibrin Gels

Fibrinogen was dissolved in PBS at 20 mg/mL, which is twice the desired final concentration. Thrombin was dissolved in PBS at a concentration of 10 U/mL. HUVECs and fibroblasts were mixed at a ratio of 5:1 and spun down at 200 rcf for 5 min. The cell pellet was resuspended in endothelial cell medium containing 2 U/mL thrombin over ice. The solution was mixed with fibrinogen solution over ice at a 1:1 ratio to produce a final fibrinogen solution with a desired concentration of 10 mg/mL and then pipetted into the PDMS devices immediately, where gelation happened within minutes. Cells were cultured using the medium described above.

### 2.8 Formation of Cell-Containing Granular Hydrogels

Microgel dilutions were achieved by centrifuging microgels alone at 21,130 rcf for 5 min in microcentrifuge tubes. We aspirated off the endothelial cell medium supernatant leaving fully jammed microgels and pipetted 100 μL of microgels into a fresh microcentrifuge tube. Desired volumes of fresh endothelial cell medium mixed with cell concentrations were added to the microgels to obtain the desired ratios of microgels:cell medium. As described above, 100 μL of microgels with 100 μL of medium gave a 1:1 ratio (the highest dilution) and 100 μL of microgels with no medium gave a 1:0 ratio, which were considered fully jammed particles. We homogeneously mixed PEG microgels with HUVECs and fibroblasts at a ratio of 5:1 in endothelial cell medium with minimal cell medium. We placed microgels and cells in the PDMS culturing device for cell culture according to the methods described above. Samples were fixed with 4% paraformaldehyde after 4 days to examine vessel networks.

### 2.9 Cell Staining

To visualize cell movement during culture, HUVECs were stained with a CellTracker Deep Red fluorescent dye (Invitrogen) in some experiments. In experiments measuring cell viability, cells were stained using a LIVE/DEAD assay kit (ThermoFisher, L3224) with calcein-AM (live cells) and ethidium homodimer-1 (dead cells) staining, and counts of live and dead cells were reported. In experiments analyzing the networks cells formed in the granular hydrogels, cocultures were fixed with 4% paraformaldehyde for 15 min before 1 h permeabilization with a 0.1% (v/v) Triton X-100 solution in PBS with bovine serum albumin (BSA, 3% (w/v)) to prevent nonspecific binding. Samples were washed with PBS and stained with primary antibodies, CD31 (Thermo Fisher, MA5-29474, 1:50 dilution), an endothelial cell marker, or anti-VE-Cadherin (Abcam, ab232880, 1:50 dilution) for endothelial cell adherens junctions for 1.5 h in blocking solution. Samples were then washed again with PBS and stained with secondary antibodies of donkey antirabbit 647 (Abcam, ab150075, 1:500 dilution) as well as phalloidin 568 (1:1000 dilution) to label F-actin. DAPI was used to illuminate cellular nuclei when the slides were mounted to the coverslip.

### 2.10 Imaging and Image Analyses

Imaging was conducted on a Leica DMi8 widefield microscope and an Olympus FV1000 confocal microscope. Images of microgels and cells were collected from devices on microscope slides. Other images containing the PEG hydrogel and cells were collected on 25 × 25 mm glass coverslips. Imaging settings (exposure time and light intensity) were consistent for all imaging, where fluorescence intensities were compared across multiple samples. At least three distinct regions of interest per scaffold were imaged for the cell morphology analyses. Images were processed by using Fiji software. Cellular connectivity was assessed computationally via REAVER^49^ to determine the cytoskeleton area fraction.

### 2.11 Statistical Analyses

Statistical analyses were performed using GraphPad Prism software. One-way analysis of variance (ANOVA) with Tukey’s multiple comparisons test (α = 0.05) was used to compute vascular formation differences between groups of microgels containing RGD, no RGD, and RDG (*n* = 3 devices per group). Preprocessing was conducted on *in vitro* cell images using the MATLAB histogram equalization function to obtain optimal visual of microvasculature networks. Preprocessed images were then analyzed using REAVER software to quantify cellular networks. One-way ANOVA with Tukey’s multiple comparisons test (α = 0.05) was used to compute differences of pore space between groups of microgel:cell media volumes (Supp. Figure 1, *n* = 3).

## 3 Results and Discussion

**Figure 1:**
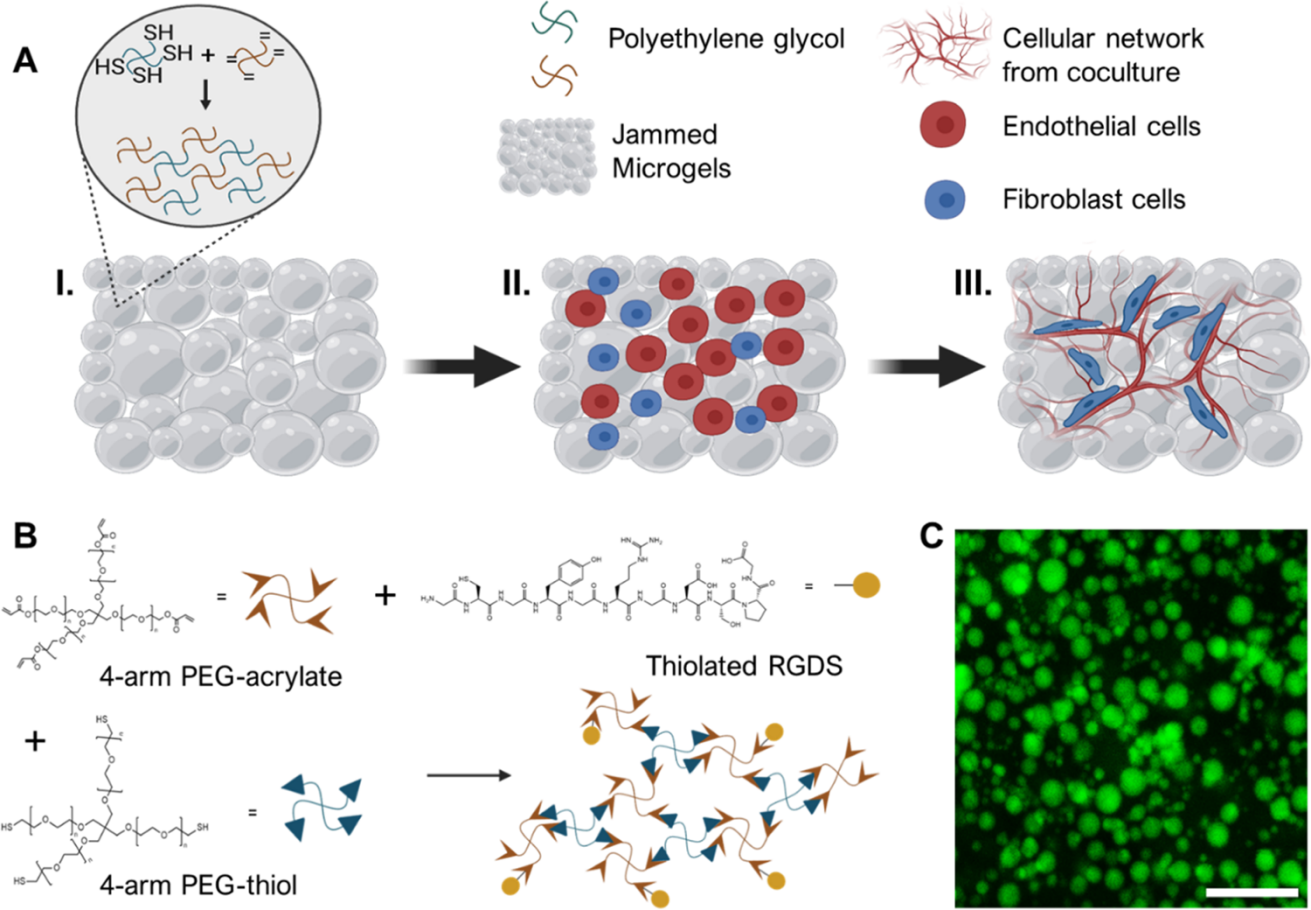
A granular hydrogel is formed from many individual PEG microgels, where excess fluid has been removed from between the microgels to result in jamming. **A.** Schematic illustration of (***I***) a granular hydrogel formed from jammed microgels (gray), where individual microgels are formed in emulsion through the cross-linking of polyethylene glycol (PEG)-acrylate and PEG-thiol (inset: hydrogel network schematic); (***II***) by including endothelial cells, a vascularized construct can be formed using endothelial cells (red) and fibroblasts (blue); (***III***) over time, cells mature into a tissue structure formed from endothelial cells supported by fibroblasts. **B.** PEG macromer and peptide chemistry, as well as hydrogel network schematic: 4-arm PEGs are combined, with acrylates reacting with thiols at elevated pH. Stoichiometric control can leave unreacted acrylates for further functionalization. **C.** Representative image of microgels (2 MDa FITC-dextran is encapsulated for visualization and size characterization) created by emulsification, dispersed in excess water before jamming (scale bar = 200 μm).

PEG hydrogels are widely used to support 3D tissue engineering, vasculogenesis,^41^ and organoid culture.^9^ To vascularize constructs or enable the morphogenesis of tissue structures within bulk PEG hydrogels, permissive chemistries are needed such as degradable cross-linking. In a granular formulation that does not contain interparticle cross-linking, we aimed to use a nondegradable PEG formulation to form microgel particles via emulsification of PEG-thiol and PEG-acrylate in solution. We hypothesized that cells introduced among the PEG microgels would be able to self-organize in a culture environment designed to support the granular hydrogel against loss (Figure 1A). We aimed to elucidate parameters that would support cell expansion and self-organization in this system.

**Figure 2:**
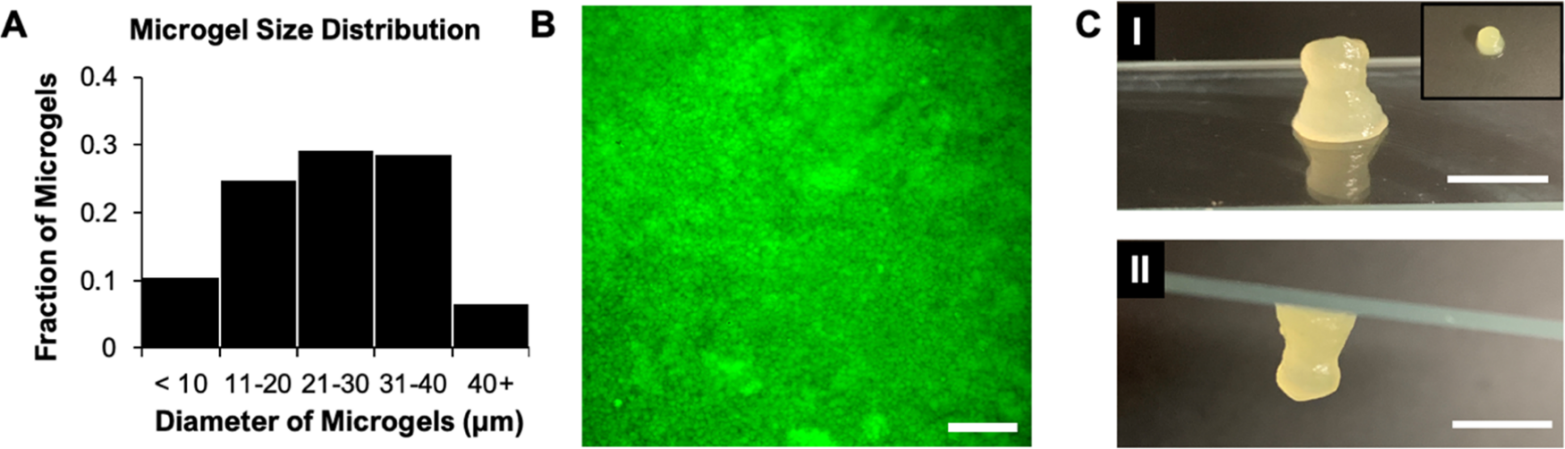
Microgel characteristics and images of a non-cross-linked granular hydrogel (microscopic and macroscopic). **A.** Representative size distribution for individual microgels within a granular hydrogel, with an average diameter of 30 μm. **B.** A microscopic image of a granular hydrogel formed by jamming of a batch of microgels (containing FITC-dextran) shows concentrated individual microgels (scalebar = 200 μm). **C.** Macroscopic images of a granular hydrogel (***I***) showing the appearance of the granular hydrogel on a glass slide (inset: top view) and (***II***) illustrating its stability even after it is inverted (scale bars = 0.5 cm). The stability of the bulk gel is due to the physical forces of jamming.

**Figure 3:**
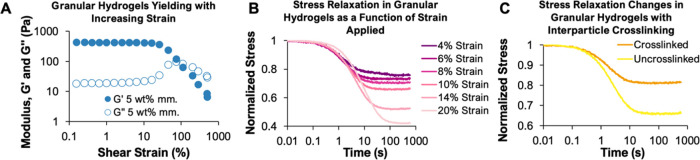
Characterization of the rheological and mechanical properties of granular hydrogels formed from PEG microgels. **A.** An oscillatory strain sweep of a 5 wt % PEG-acrylate^/^PEG-thiol granular hydrogel exhibits elastic behavior below a strain at which the yield stress is exceeded, resulting in fluid-like behavior. **B.** Stress relaxation of 5 wt % granular hydrogels after applying various strains. The granular hydrogels exhibit increasing stress relaxation times as strain increases with final stress plateauing at a lower percent of the maximum stress. **C.** Stress relaxation in granular hydrogels varies as a function of forming cross-links between particles within the granular material. Interparticle cross-linking decreases the gels’ ability to relax, resulting in higher stresses in the hydrogels after relaxation compared to un-cross-linked granular materials.

PEG microgels were produced via cross-linking of acrylate- and thiol-functionalized PEG by Michael addition in emulsion. Excess acrylate groups were available for subsequent conjugation to cysteine-containing peptides such as the fibronectin-derived cell-adhesive ligand RGD (Figure 1B). To visualize PEG microgels, high-molecular-weight (2 MDa) fluorescein-isothiocyanate-dextran was included within the PEG solution during cross-linking. After recovery from oil, washing to remove surfactants, and reswelling in excess water, PEG microgels (Figure 1C) were found to have a size distribution where particle sizes were <40 μm diameter (Figure 2A). These microgel sizes were chosen to approach a size similar to that of cells to facilitate a more homogeneous distribution of cells within the volume of the granular system and, potentially, to create particles that might be easily displaced or traversed by cells. A granular hydrogel formed by centrifuging a suspension of microgels as described above was observed to consist of densely packed particles (Figure 2B). This granular hydrogel formulation (Figure 2C, I), which includes no interparticle cross-linking, maintained its shape after inversion (Figure 2C, II), and displayed rheological properties similar to those seen in granular microgel systems formed from monodisperse particle populations.^48^ The physical properties of the material that are important to application and cell culture were characterized through rheological measurements on the bulk granular hydrogel. Because this granular PEG hydrogel did not include interparticle cross-linking, it maintained the ability to yield under sufficient applied strain (Figure 3A). At low strains, the granular hydrogel showed storage and loss moduli of 420 and 18 Pa, respectively, within the range typically employed in hydrogels used for soft tissue engineering.^50^ In response to increasing strains on the granular hydrogel, the yield stress was exceeded and the granular hydrogel switched from solid-like to fluid-like behaviors (Figure 3A). This behavior is characteristic of granular hydrogels in which physical jamming interactions, but not interparticle cross-linking, stabilize the bulk materials.^50^ These properties were also seen in the diluted hydrogel formulations used in cell studies. Despite increasing porosity, particle–particle interactions stabilized the bulk material at low strains, where it exhibited solid-like behavior, with yielding occurring at increased strain (Supp. Figure 2).

Stress relaxation is characteristic of biological matter, which can rearrange over time to dissipate a force resulting, for example, from a sustained strain applied to the material.^34^ Toward designing biomaterials with properties that match tissue, the ability to engineer stress relaxation would be advantageous. At the scale of cellular interactions with the extracellular environment, the ability of a material to relax in response to cellular activity is important for cellular processes that include cell migration,^34^ cellular^38^ and tissue morphogenesis,^9,20,51^ and vasculogenesis.^20^ Here, we looked to measure bulk stress relaxation in a granular hydrogel, which we expected to be enhanced by the absence of interparticle cross-linking. Importantly, while local stress relaxation in response to cellular activity might enhance permissiveness and facilitate cell migration and self-organization, as mentioned above, the jammed nature of the granular system was viewed as unlikely to permit significant cell-driven reorganization of microgel particles. It is possible that certain particle sizes and stiffnesses might be used to establish environments that yield to the activities and forces exerted by single cells, as has been demonstrated using ultrasoft particles^39^ and in nanofiber-based systems.^52,53^

To quantify the bulk stress relaxation of the granular system, we used rheology to apply increasing strains and measured the stress responses. Under increasing strains, the granular hydrogel exhibited increasing stress relaxation, as measured relative to the initial stress applied. Stress is relaxed on the order of 10 s of seconds within these systems, with longer relaxation times at higher strains reflecting larger applied stresses requiring more time reorganization of the granular material to relax (Figure 3B). At higher strains, the stress supported by the granular material does not relax to zero due to jamming of the elastic microgels supporting a yield stress, as shown in Figure 3A. Once applied stress no longer exceeds the forces required to cause flow within the granular system, the bulk material properties again become similar to those of an elastic hydrogel. To quantitatively show that eliminating interparticle cross-linking enhanced stress relaxation within the granular hydrogel, we applied a 10% strain to both the un-cross-linked and cross-linked granular hydrogels and observed the stress relaxation response (Figure 3C). We observed a decreased ability to relax stress in granular materials where there was particle-to-particle cross-linking, resulting in higher sustained stresses after relaxation in cross-linked granular hydrogels compared to un-cross-linked granular hydrogels where particle flow was restricted solely by physical jamming interactions.^6,100,54^

At the cell scale, fate processes such as migration, proliferation, and differentiation are influenced by material features such as porosity^31^ that promotes a permissive environment, as well as growth factors,^6,40,53^ and cell-adhesive ligands.^41,55^ In this work, we were interested in developing a system that would harness the inherent porosity of a granular hydrogel without interparticle cross-linking in a system that would allow us to modify adhesive ligands and apply growth factors in culture. Porosity in granular hydrogels can facilitate vascularization by promoting cellular ingrowth both *in vivo*(31) and *in vitro.*(52,53,56) To control porosity in our system, we looked to decrease microgels packing by dilution to increase porous spaces (fully jammed microgels (1:0) to 1 part medium to 1 part microgels (1:1)) and reduce microgel contacts (Supp. Figure 1). Changes to the physical environment through increased porosity and reduced jamming should enhance cells’ abilities to self-organize within their material surroundings. In all 3D cell culture experiments, we incorporated a coculture of HUVECs and 3T3 fibroblasts that were known to self-organize into network structures, including microvascular structures, under the appropriate conditions. Cell types were chosen due to their known behavior in engineered tissue systems and models of microvasculature. In engineered tissues, 3T3 fibroblasts will expand within spaces and deposit extracellular matrix and HUVECs will establish microvascular networks, supported by the presence of 3T3s and related signaling.^57^ This coculture was thus selected for use in experiments where we desired to observe the emergence of multicellular architectures given that it was known to exhibit self-organizing behavior.

**Figure 4:**
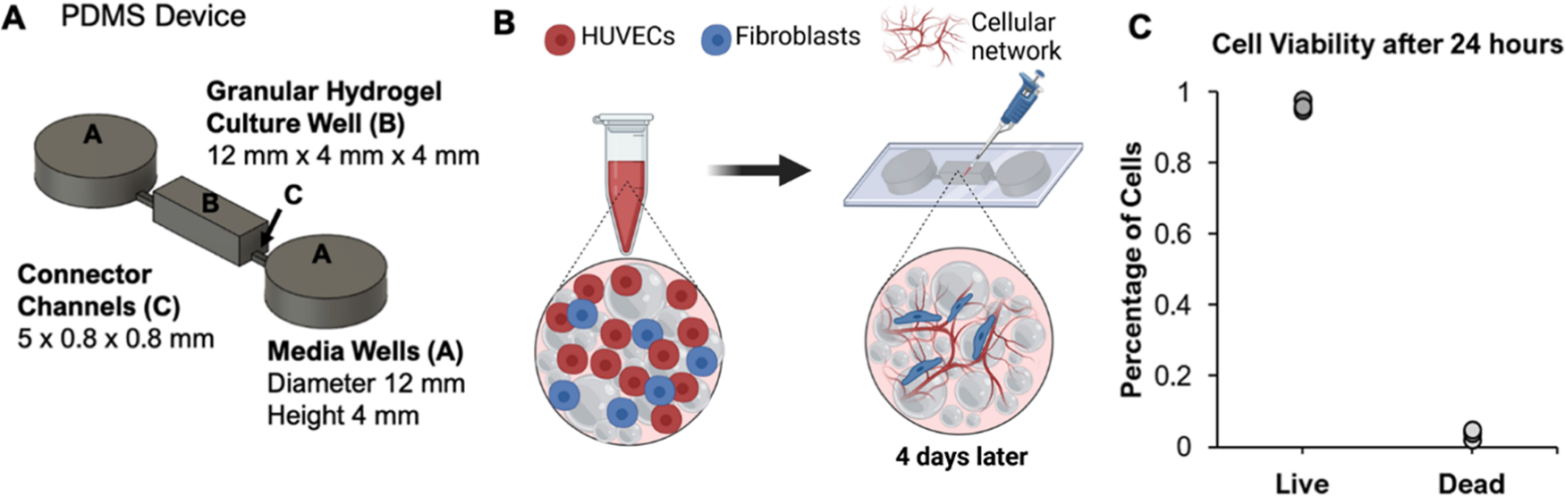
3D culture experiments were performed in PDMS microdevices designed to hold the granular hydrogels in place, absent interparticle cross-linking. **A.** The 3D computer design of the PDMS mold is shown with the gray volumes representing the void space in the final device. The final device includes two cell medium wells with 12 mm diameters, a rectangular central well for granular hydrogel culture, and two connector channels for medium perfusion and nutrient/waste exchange. **B.** Schematic of coculture experiment with microgels. HUVECs and 3T3 fibroblasts were mixed with PEG microgels to form a homogeneous mixture, which was then pipetted into a center culture well in the PDMS device. Endothelial cell medium was applied to the center well from the two outer circular wells via connector channels. **C.** Cell viability was quantified after 24 h: >96% viability was observed.

To facilitate cell culture in granular systems that did not have interparticle cross-linking, we created a custom polydimethylsiloxane (PDMS) device for coculture (Figure 4A,B). To confirm cell viability within the device, we introduced into the central well of the device the HUVEC/fibroblasts coculture within the fully jammed microgels, which were modified to include RGD. Cell survival 24 h post seeding was observed using a Live/Dead assay. Cell viability within the granular hydrogel was greater than 96% 24 h post seeding into PDMS devices (Figure 4C and Supp. Figure 3).

**Figure 5:**
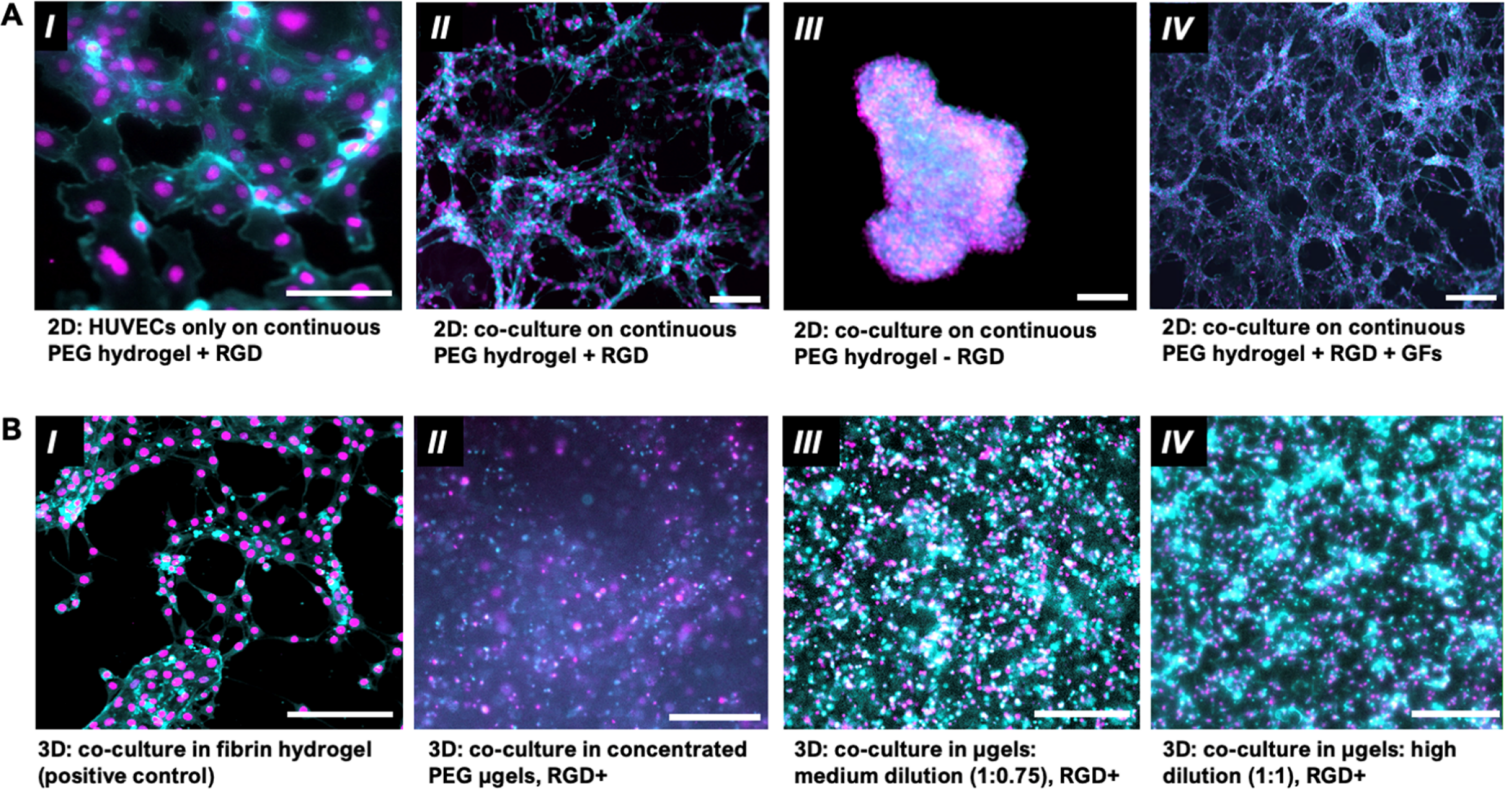
Emergence of self-organized structures was observed in 2D PEG hydrogels, in a 3D fibrin hydrogel (as a control), and in 3D granular hydrogels formed by using PEG-based microgels. F-actin staining (cyan) and nuclear staining (magenta) are shown. **A.** Bright-field images of (***I***) HUVEC monoculture on the surface (2D) of a continuous PEG hydrogel where RGD is added (+RGD) exhibits cobblestone-like cell morphologies reminiscent of blood vessels’ endothelial lining. (***II***) Coculture of HUVECs with 3T3 fibroblasts on the surface of a continuous PEG hydrogel (+RGD) results in the organization of endothelial cells into a connected network. (***III***) In the absence of RGD, cells cluster instead of self-organizing. (***IV***) The addition of growth factors to the culture medium (+GFs) enhances cellular network formation in cocultures of HUVECs and 3T3 fibroblasts on 2D continuous PEG hydrogel (+RGD) surfaces. **B.** A fibrin hydrogel was used (***I***) as a control to confirm the organization of cellular network structures in 3D coculture from HUVECs and 3T3 fibroblasts that were homogeneously dispersed at the start of the experiment, as has been shown in previous work.^40^ (***II***) In 3D cultures within PEG-based granular hydrogels that include RGD, cellular network formation is seen to be enhanced with increasing dilutions of the microgels in the granular hydrogel, from fully jammed, to (***III***) a 1:0.75 dilution of the microgels (combining 1 part jammed μgels with 0.75 parts medium), to (***IV***) a 1:1 dilution of microgels with cell medium. All scale bars = 200 μm.

To confirm culture conditions that support HUVEC and fibroblast coculture’s self-organization into network structures, we monitored the formation of cellular networks in 2D and 3D nongranular control systems. First, HUVECs labeled with CellTracker (dsRed) were cultured together with fibroblasts on transwell inserts, in a well-established transwell system.^58^ After 24 h, we observed the expected organization of endothelial cell networks (Supp. Figure 4A) with continued maturation after 4 days observed through VE-Cadherin staining (Supp. Figure 4B). Next, the emergence of cellular networks was observed on 2D hydrogel surfaces (Figure 5A). On the surface of a 2D PEG hydrogel, the inclusion of RGD and of fibroblasts in coculture with HUVECs was necessary to drive network formation. We observed that on a 2D PEG hydrogel with RGD, when HUVECs are cultured alone, they form a cobblestone-like structure on the hydrogel surface with RGD, reminiscent of their roles in creating a monolayer lining the vascular wall (Figure 5A, I). The introduction of a supporting cell type, fibroblasts, induced the formation of microvessel-like structures (Figure 5A, II), while hydrogels without RGD prevented network formation and resulted in cell aggregation (Figure 5A, III). Next, we wanted to test if the addition of growth factors (100 ng/mL human angiopotetin-1 protein and 50 ng/mL VEGF-165A)^6,40,101^ to the culture medium decreased time to achieve microvessel-like connectivity and branching. After 4 days, 2D culture in the presence of growth factors on a continuous, RGD-modified PEG hydrogel supported more extensive network coverage (Figure 5A, IV) compared to culture conditions without supplemented growth factors (Figure 5A, II).

To observe network formation in the 3D hydrogels (Figure 5B), we first used fibrin hydrogels as a positive control. Fibrin hydrogels have been well-documented supporting robust cellular networks from 3D cocultures of HUVECs and fibroblasts.^40^ From cells dispersed homogeneously in a fibrin gel, we observed the emergence of cellular network structures over 4 days, confirming our coculture, both the HUVEC/fibroblast concentration and ratio, and medium formulations with growth factors supported cellular self-organization in a permissive 3D material environment (Figure 5B, I).

To assess whether granular formulations of a nondegradable PEG hydrogel in which there was no interparticle cross-linking would support cellular self-organization in 3D, we next cultured HUVECs and fibroblasts among PEG microgels within the PDMS device (Figure 4B). Using the coculture conditions established for fibrin hydrogels,^40^ we looked for the development of self-organized cellular structures from a homogeneous dispersion in 3D. Over 4 days in our fully jammed system, cells were observed to assemble into cell-dense regions. However, the multicellular structures did not appear to exhibit any network-like connectivity (Figure 5B, II). We next looked to decrease microgel packing by dilution to increase porous spaces and reduce microgel contacts (Supp. Figure 1). Changing cells’ physical environment through increased porosity and reduced jamming enhances their ability to move within their material surroundings. By diluting the microgel density in the granular hydrogel with either 0.75 parts medium to 1 part fully jammed microgels or 1 part medium to 1 part microgels, we observed cellular networks via actin staining (Figure 5B, III and IV) that more closely resembled the self-organized structures in the fibrin hydrogels (Figure 5B, I). From this experiment, it was evident that within a granular material environment, increasing porosity and decreasing particle contacts supported cells establishing connectivity. In comparison, densely jammed particles appeared to limit cell–cell interactions and rearrangements despite exhibiting macroscale stress yielding and relaxation.

**Figure 6:**
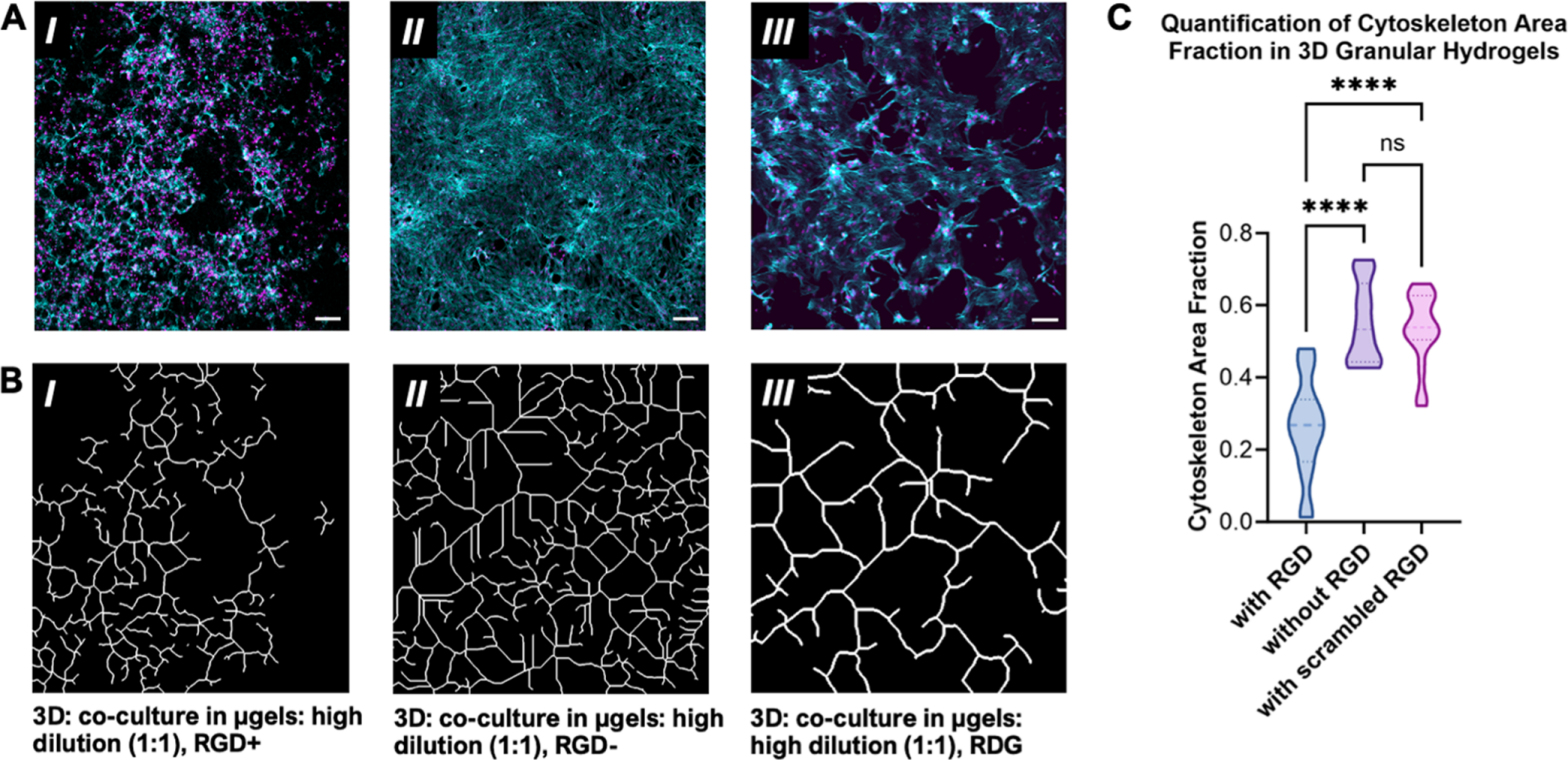
Cellular networks formed from HUVECs and 3T3 fibroblasts in 3D granular PEG hydrogels responded to the inclusion of the cell-adhesive ligand RGD (magenta: DAPI; cyan: actin). **A.** Confocal image after 4 days (***I***) exhibits cellular connectivity in the presence of RGD (RGD+) and (***II***) more expansive cell morphologies and networks in the absence of RGD (RGD−). In the presence of scrambled RGD (RDG) (***III***), cellular morphologies are extended. All scale bars = 200 μm. **B.** Confocal images were assessed computationally via REAVER to obtain the wire frame of cytoskeleton in the presence of RGD (RGD+) (***I***), absence of RGD (RGD−) (***II***), and presence of scrambled RGD (RDG) (***III***). **C.** REAVER quantification of cytoskeleton area fraction in all groups was assessed. One-way ANOVA with Tukey’s multiple comparisons test showed significant differences between ratio RGD+ and RGD– (****P < 0.0001) and RGD+ and RDG (**P < 0.01). No significant differences between RGD– and RDG were observed with respect to area of the cytoskeletal coverage (ns: *P* > 0.05).

Finally, we examined the influence of cell adhesion to microgels in 3D granular systems on the cellular self-organization. In 2D PEG systems, cell-adhesive ligands (RGD) support the formation of cellular networks (Figure 5A), however in 3D, we observed the emergence of multicellular structures in the absence of RGD (Figure 5B). In contrast, RGD was either natively present^101−60^ or added to other 3D hydrogel systems designed to support angiogenesis^19,55^ and vasculogenesis^20^ in nongranular hydrogels. In our granular PEG hydrogels, we thus compared the emergence of cellular structures in granular PEG hydrogels that included or did not include PEG-tethered RGD ligands. Here, using our most dilute granular PEG hydrogel for experimentation (1:1 PEG:medium), we observed that when RGD was present, cells appeared to organize into network-like cellular structures (Figure 6A,B, I and Supp. Figure 5A,B). However, when we removed RGD from our PEG microgels, we consistently observed more extensive cellularity and network formation (Figure 6A,B, II and Supp. Figure 5A) compared to when RGD was present (Supp. Figure 5B). In all conditions, cellular networks formed within the 3D space of the granular system (Supp. Figure 6) and staining for CD31 and VE-cadherin evidenced the presence of endothelial cells within these networks (Supp. Figure 7).

To confirm that the observed effect of hindered network formation was specifically due to RGD and not more generally due to the surfaces of PEG microgels being modified with a peptide, we formulated PEG microgels that presented RDG, which is a scrambled version of the RGD peptide. Here, we observed an effect similar to when PEG microgels did not contain RGD. When microgels are developed with RGD, decreased microvasculature networks (Figure 6A,B, I) are observed compared to those of microgels without RGD (Figure 6A,B, II) and microgels with RDG (Figure 6A,B, III). Network coverage was quantified computationally using MATLAB preprocessing and REAVER,^49^ a software tool established for analyzing network structures. We found there was roughly 30–40% cytoskeleton area in microgels containing RGD after 4 days in cocultures (Figure 6C), compared to a roughly 60% cytoskeleton area fraction with microgels in the absence of RGD and in the presence of RDG. This result was unexpected considering previous work in continuous hydrogels focused on developing microvasculature structures. The increased cellularity in the absence of RGD suggests that within the permissive environment of a granular hydrogel, alterations in cell-hydrogel interactions affect cellular response and drive differential self-organization responses in 3D. It is probable that the removal of RGD effectively removed the potential for cell-matrix interactions and altered processes relevant to self-organization in this coculture. As a result of changes to signaling pathways that are induced by integrin engagement of RGD, the differential response observed might be a result of changes in cell–cell interactions or behaviors in the coculture, as cells within systems that did not include RGD appeared more spread in actin staining. It is possible that changes to actin organization reflect a difference in migratory state, with cells being less migratory with more static and extended cytoskeletons in the absence of RGD.^61^ Similar results were observed when PEG microgels contained a scrambled RGD peptide, RDG (Figure 6C).

**Figure 7:**
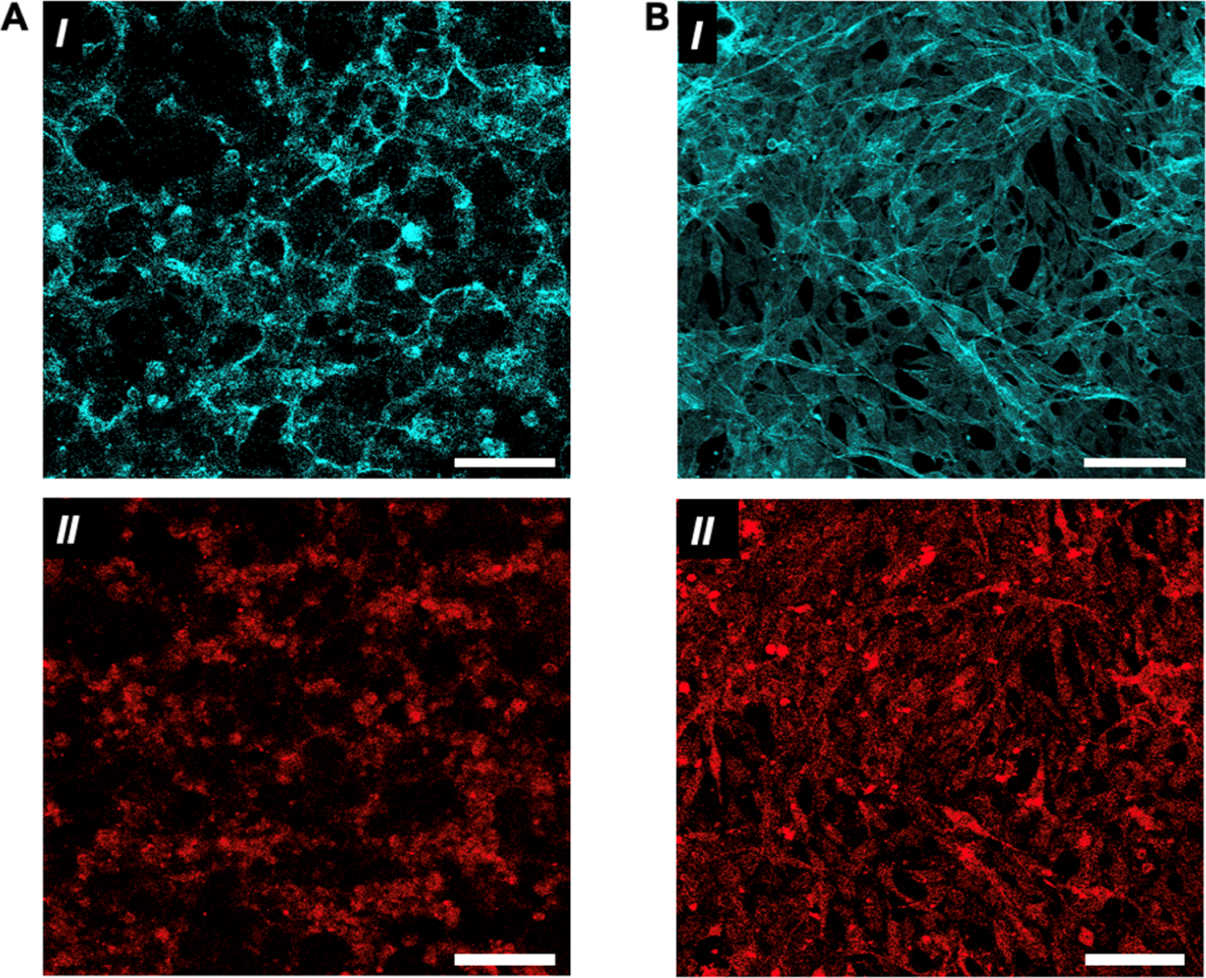
Confocal imaging to confirm endothelial cell networks formed from cocultures of HUVECs with 3T3 fibroblasts, staining for actin (cyan) and CD31 (red), an endothelial cell marker. **A.** After 4 days, cells in the presence of RGD exhibit cellular connectivity (***I***) with endothelial cells throughout the network (***II***). **B.** In the absence of RGD, cellular connectivity (***I***) and endothelial cells are similarly seen throughout the network, with extended morphologies observed (***II***). All scale bars = 100 μm.

Because our coculture contained both HUVECs and fibroblasts, and the changes in morphologies suggested the possibility of enhanced fibroblast activity, we wanted to assess the presence of HUVECs through staining of endothelial cell marker, CD31 (PECAM-1) (Figure 7A,B). In both the presence of RGD and the absence of RGD, CD31-positive staining (Figure 7A,B, II) indicates HUVECs contributing to the multicellular structures observed. The more extended cytoskeletal structures seen in actin staining in the presence of RGD (Figure 7B, I) were accompanied by CD31 staining, which suggested the endothelial cells within the system also exhibited more stretched morphology (Figure 7B, II). In the absence of RGD, the cells interrogate the 3D environment without ligating matrix-bound peptides, which may lead to increased cell–cell interactions—in the absence of competing matrix-tethered signals—and stiffening through interactions with neighboring cells or deposited ECM, as indicated by the presence of stress fibers in the actin staining.^62^ In other words, absent RGD, HUVECs may receive a higher proportion of instructive cues from fibroblasts, for example, through direct cell–cell interactions or matrix deposition.

Here we studied the presence or absence of RGD and its effects on cellular behavior at a single RGD concentration. RGD concentration was selected to provide a comparison to a 2D system where RGD was necessary for network formation (Figure 5). The role of RGD in a 3D environment is complex and while, in the absence of other ligands for mediating cellular traction, it may be necessary to drive fate decisions, other ligands or ligand concentrations might be better suited to specific applications.^63−65^ For example, peptide mimetics of protein binding sites in laminin, collagen, or fibronectin, or full-length proteins, might be studied with respect to effects on the emergence of multicellular structures. Additionally, protein deposition by cells, altering their interactions with an engineered matrix, could be expected to influence cell behavior at extended time points. The observed effects of RGD here, in which it is observed to decrease network connectivity, a result that is reversed with the peptide being scrambled, might be further studied in terms of additional nonmorphological cellular responses to RGD concentration, matrix mechanical properties, and other tethered signals in the context of a given application. Additionally, forming microgels from gelatin or other natural or ECM-derived materials that contain intrinsic cell adhesion domains, could be expected to provide complex signals to cells that support the formation of tissue structures without adhesive peptides.^12,40,66^

These results open directions for further study toward specific cellular processes, such as vasculogenesis or maturation of multicellular assemblies in un-cross-linked systems. Cellular organization might be further enhanced by decreasing jamming and through the design of integrin-binding ligands within the hydrogel surroundings. The cell-scale (<40 μm diameter) PEG microgels used here might be further engineered toward various applications through biofunctionalization and changes in polymer concentration or intraparticle cross-link design.

## 4 Conclusions

Here, we have established permissive environments in synthetic granular hydrogels in which there is no interparticle cross-linking, which allows bulk relaxation and eliminates covalent interactions that might restrict cellular activity. Both in the presence and absence of a traditional cell-adhesive ligand, RGD, we see that increased permissivity gives rise to cellular self-organization. We show that cellular networks can form rapidly within a permissive environment, on time scales that are fast and cell seeding densities that are low compared to cellular network formation in vasculogenic processes in hydrogel systems.^32,67^ As is appreciated from the study of complex 3D biomaterial systems, we see evidence for differential contributions of ligand binding, cell–cell interactions, material viscoelasticity, and permissivity (yielding or degradation) depending on system design. Specific cellular processes under consideration may influence the design. For example, in contrast to our results, RGD might be necessary for angiogenic processes where cells are not embedded within a material but instead infiltrate into the granular system to form vessels. The importance of enzymatic^20^ activity, or of the ability of a polymeric network to yield and be reorganized, which is crucial to cellular self-organization in continuous 3D hydrogels, may be less important in a granular material. In a granular material, we show that enhanced permissivity by reducing jamming concentration might have multiple effects: both allowing space and reducing particle–particle contacts and physical interactions that hold microgels in place in jammed granular systems. This work demonstrates that un-cross-linked 3D granular systems can support robust cellular activity and self-organization. We expect that approaches to granular materials that reduce physical interactions, such as eliminating cross-linking, may ultimately facilitate displacement of particles by cells^35^ and support tissue biofabrication processes that would not be possible in fully cross-linked systems. This work demonstrates that stress-relaxing granular biomaterials that are composed of covalently cross-linked microgels, but no particle-to-particle cross-linking can support the maturation of multicellular structures. These observations may have implications for understanding how complex tissue structures evolve and expand approaches for supporting their engineering in 3D biomaterials.

## Supporting Information Available

Thresholded images of varied microgel pore space and quantification (S1); rheological measurements of varied microgel pore space (S2); bright-field image of LIVE/DEAD within granular hydrogel system (S3); fluorescent images of cells only seeded on transwells after 24 h and 4 days (S4); fluorescent images of coculture environment of HUVECs and fibroblasts within microgel scaffold (S5); 3D images of cellular networks within granular hydrogel systems (S6); and fluorescent images of CD31 and VE-Cadherin staining within granular hydrogel systems (S7) (PDF)
