# Supplementary material for: Engineering Granular Hydrogels without Interparticle Cross-Linking to Support Multicellular Organization
Source: ACS Biomater Sci Eng. 2024 Nov 25;10(12):7594–605. doi: 10.1021/acsbiomaterials.4c01563 (PMC11632665; doi:10.1021/acsbiomaterials.4c01563)
Supplement: Supplementary file 1 — ab4c01563_si_001.pdf [file ab4c01563_si_001.pdf]

Engineering Granular Hydrogels Without Interparticle Crosslinking to Support Multicellular Organization

Natasha L. Claxton[†], Melissa A. Luse[#], Brant E. Isakson[#,‡], and Christopher B. Highley[†,¥*]

[†]Department of Biomedical Engineering, University of Virginia, Charlottesville, VA 22903, USA
[#]Department of Molecular Physiology and Biophysics, University of Virginia School of Medicine, Charlottesville, VA 22903, USA
[‡]Robert M. Berne Cardiovascular Research Center, University of Virginia School of Medicine, Charlottesville, VA 22903
[¥]Department of Chemical Engineering, University of Virginia, Charlottesville, VA 22903, USA

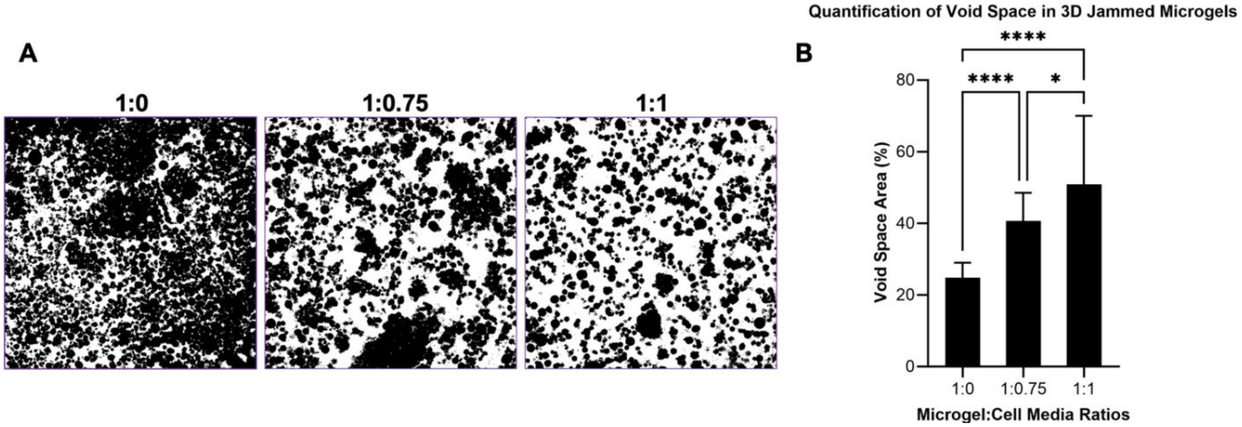

Supplementary Figure 1: A. Thresholded images processed in FIJI of varying pore space of the microgel scaffolds. Black denotes microgels and white represents void space in the scaffold. **B.** Quantification of pore space within microgel scaffolds. Pore space increases with microgel to cell medium volume ratio. One-way ANOVA with Tukey's multiple comparisons test showed significant differences between ratio 1:0 and 1:1, 1:0 and 1:0.75, 1:0.75 and 1:1 (*P < 0.05, ****P<0.001).

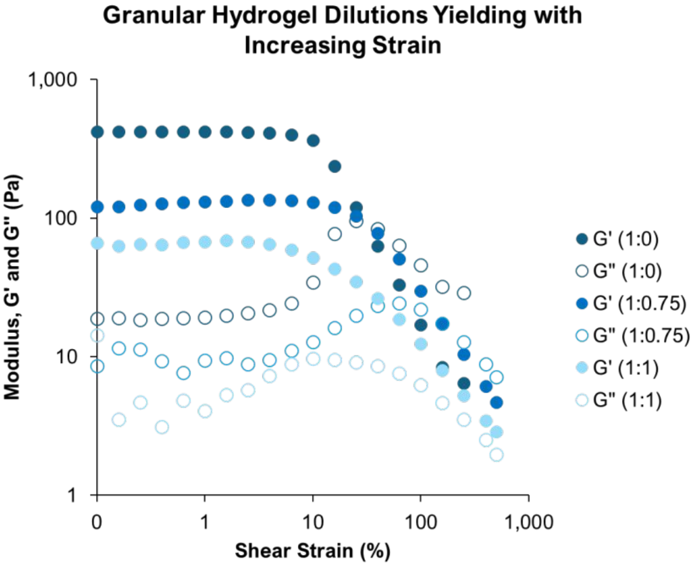

Supplementary Figure 2: Granular hydrogels with increasing dilution (pore space) exhibit bulk elastic behaviors at low strain and a transition to liquid-like behavior at high strain, similar to the fully jammed (undiluted) granular system. The dilutions here were used in cell experimentation and exhibit stable gels under static conditions, despite increasing porosity. Bulk storage and loss moduli decrease for the system as expected.

Engineering Granular Hydrogels Without Interparticle Crosslinking to Support Multicellular Organization

Natasha L. Claxton[†], Melissa A. Luse[#], Brant E. Isakson[#,‡], and Christopher B. Highley[†,¥*]

[†]Department of Biomedical Engineering, University of Virginia, Charlottesville, VA 22903, USA
[#]Department of Molecular Physiology and Biophysics, University of Virginia School of Medicine, Charlottesville, VA 22903, USA
[‡]Robert M. Berne Cardiovascular Research Center, University of Virginia School of Medicine, Charlottesville, VA 22903
[¥]Department of Chemical Engineering, University of Virginia, Charlottesville, VA 22903, USA

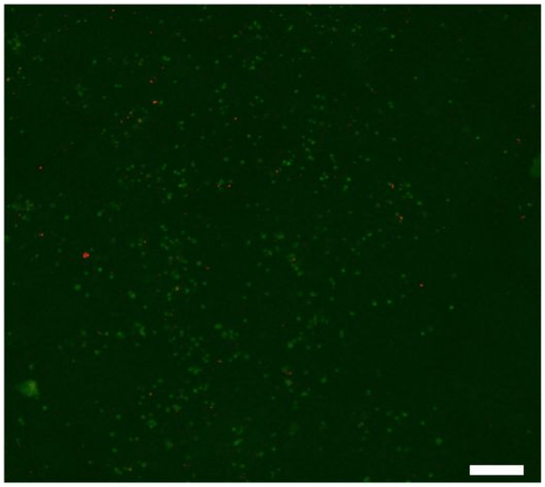

Supplementary Figure 3: Bright field of live/dead assay of cells within granular hydrogel system in device, with Calcien-AM staining for live cells (green) and ethidium homodimer-1 for dead cells (red). Scale bar = 100 μm.

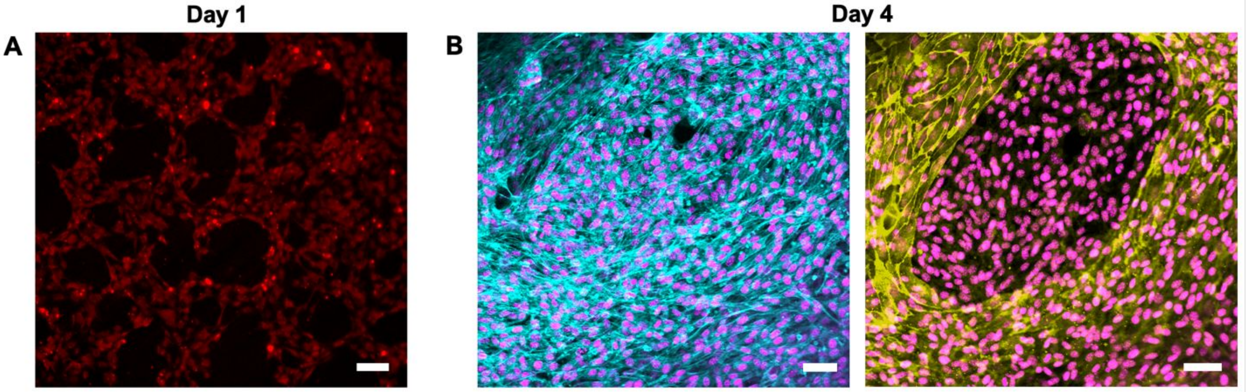

Supplementary Figure 4: A. Bright field images of cells only seeded on transwells after 24 hours with HUVECs stained with dsRed cell tracker dye. **B.** Confocal images after 4 days of co-culture where HUVECs form lumen-like structure in yellow (Vascular endothelial-cadherin (VE-Cad)) {magenta: DAPI, and cyan: actin}. All scalebars = 50 μm.

Engineering Granular Hydrogels Without Interparticle Crosslinking to Support Multicellular Organization

Natasha L. Claxton[†], Melissa A. Luse[#], Brant E. Isakson[#,‡], and Christopher B. Highley[†,¥*]

[†]Department of Biomedical Engineering, University of Virginia, Charlottesville, VA 22903, USA
[#]Department of Molecular Physiology and Biophysics, University of Virginia School of Medicine, Charlottesville, VA 22903, USA
[‡]Robert M. Berne Cardiovascular Research Center, University of Virginia School of Medicine, Charlottesville, VA 22903
[¥]Department of Chemical Engineering, University of Virginia, Charlottesville, VA 22903, USA

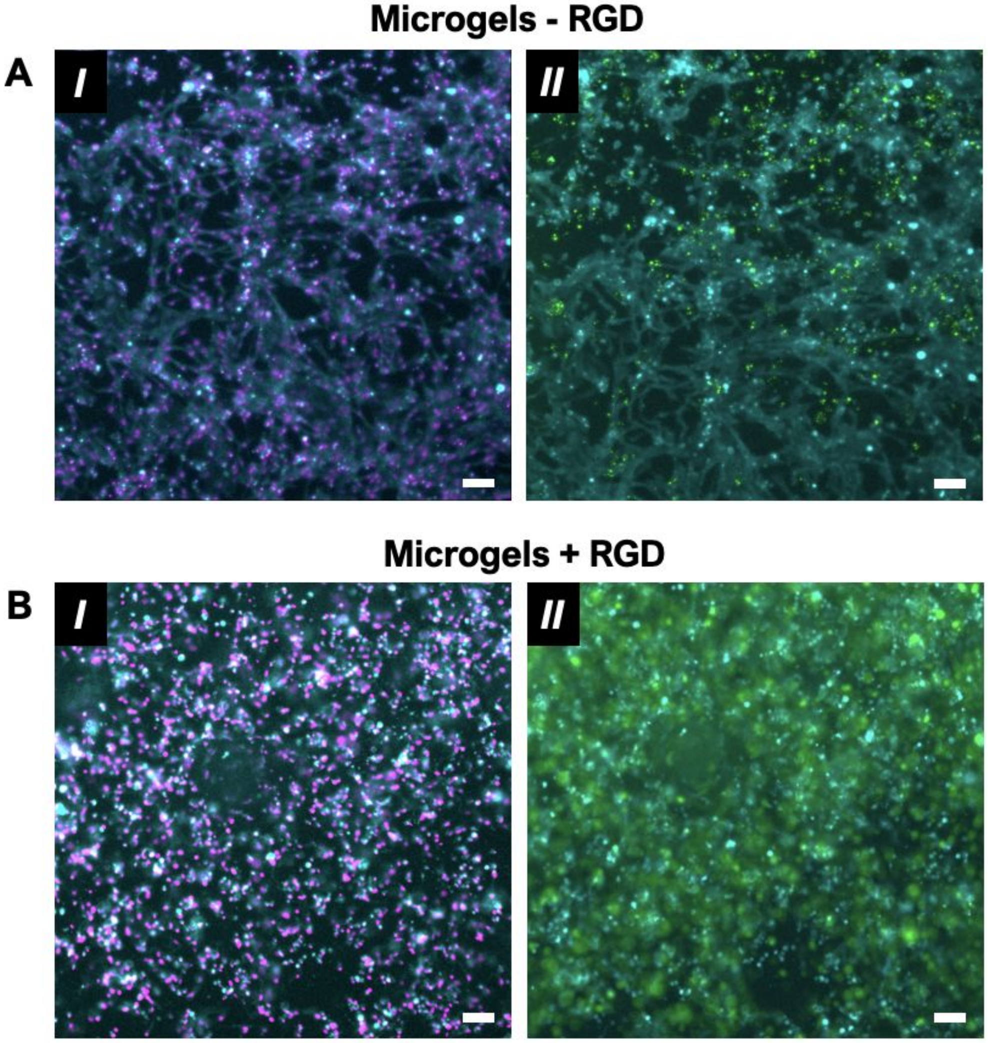

Supplementary Figure 5: Co-culture environment of HUVECs and fibroblasts within microgel scaffold. **A.** Fluorescent images after 24 hours exhibit cellular networks (*I*) in microgels without RGD (*II*). **B.** Fluorescent images after 24 hours exhibit little to no cellular connectivity formation (*I*) in microgels with RGD (*II*). Images stained for cyan: actin, magenta: cell nuclei, and green: microgels. All scalebars = 50 μm.

Engineering Granular Hydrogels Without Interparticle Crosslinking to Support Multicellular Organization

Natasha L. Claxton[†], Melissa A. Luse[#], Brant E. Isakson[#,‡], and Christopher B. Highley[†,¥*]

[†]Department of Biomedical Engineering, University of Virginia, Charlottesville, VA 22903, USA
[#]Department of Molecular Physiology and Biophysics, University of Virginia School of Medicine, Charlottesville, VA 22903, USA
[‡]Robert M. Berne Cardiovascular Research Center, University of Virginia School of Medicine, Charlottesville, VA 22903
[¥]Department of Chemical Engineering, University of Virginia, Charlottesville, VA 22903, USA

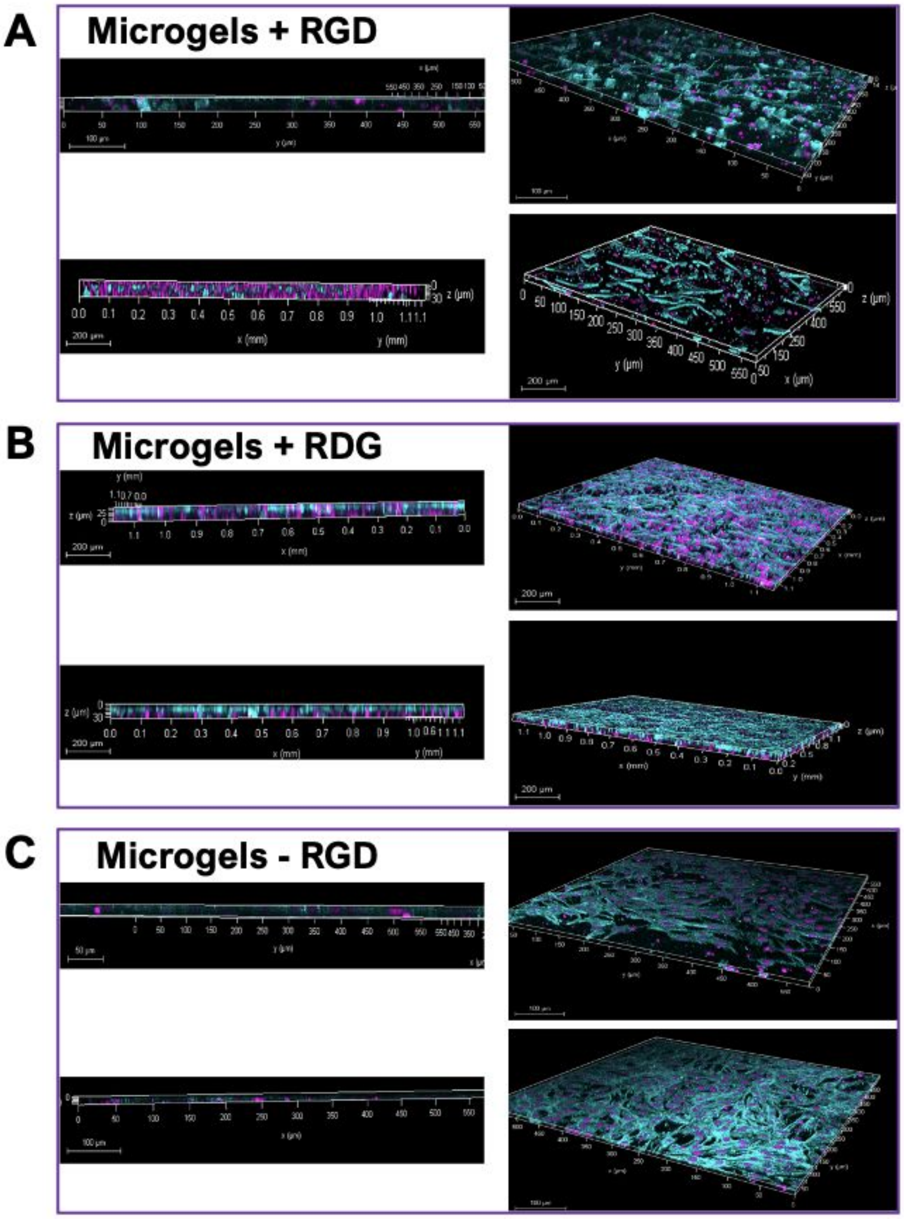

Supplementary Figure 6: Cellular networks exhibit three-dimensionality in granular hydrogel systems. **A.** Cells in granular hydrogels containing RGD exhibit sparse network structures. **B**. In granular hydrogels containing RDG (the scrambled RGD sequence) or **C**. no RGD, cellularity is increased and more extensive within the 3D granular system. Images stained for cyan: actin and magenta: cell nuclei.

Engineering Granular Hydrogels Without Interparticle Crosslinking to Support Multicellular Organization

Natasha L. Claxton[†], Melissa A. Luse[#], Brant E. Isakson[#,‡], and Christopher B. Highley[†,¥*]

[†]Department of Biomedical Engineering, University of Virginia, Charlottesville, VA 22903, USA
[#]Department of Molecular Physiology and Biophysics, University of Virginia School of Medicine, Charlottesville, VA 22903, USA
[‡]Robert M. Berne Cardiovascular Research Center, University of Virginia School of Medicine, Charlottesville, VA 22903
[¥]Department of Chemical Engineering, University of Virginia, Charlottesville, VA 22903, USA

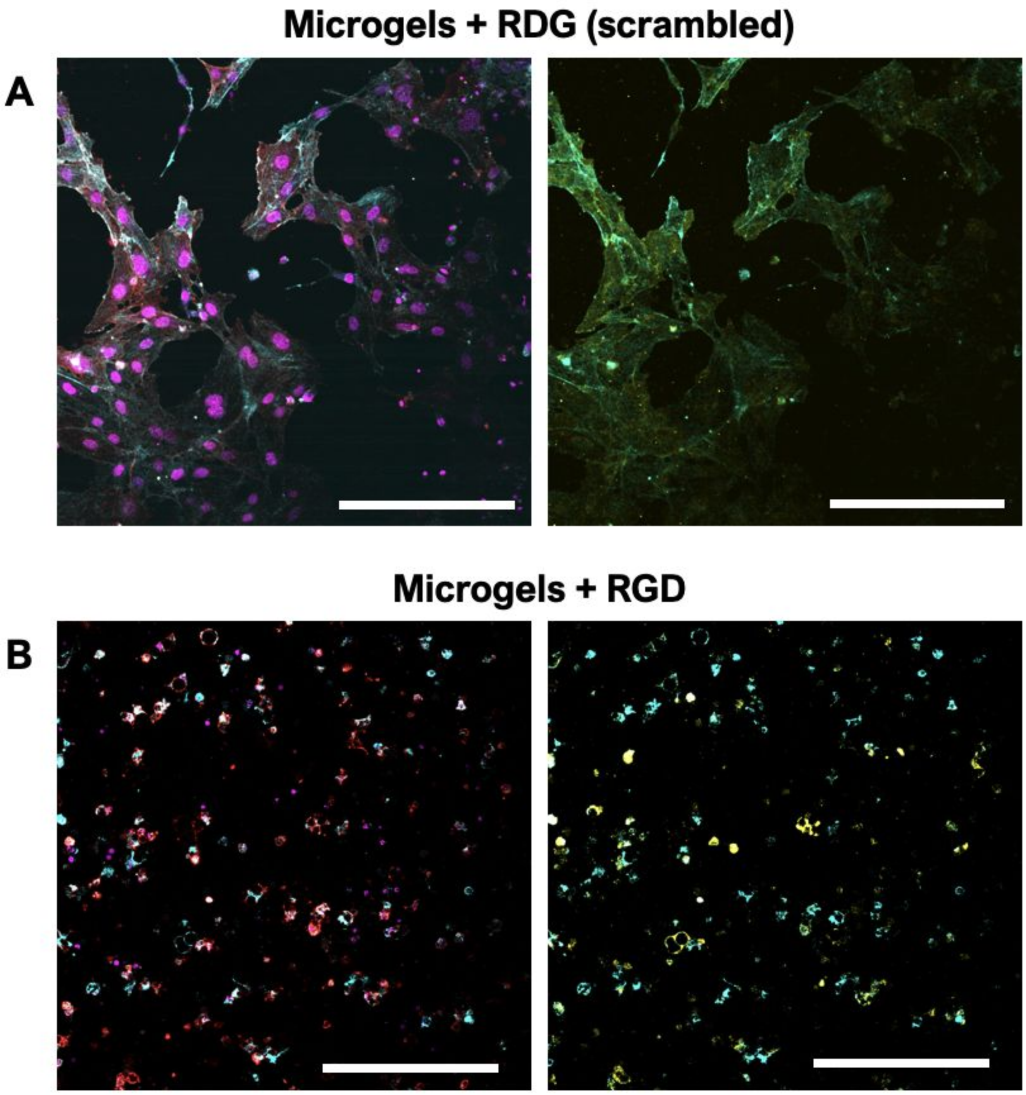

Supplementary Figure 7: Staining for markers of endothelial cells (CD31 and VE-cadherin) is evident in both **A.** the granular hydrogel containing RDG (scrambled) and **B.** the granular hydrogel containing RGD, despite differences in cell morphologies. Images stained for cyan: actin, magenta: cell nuclei, red: CD31, and yellow: VE-Cadherin. All scalebars = 100 μm.
